# Towards the expressive power of translating approach for knowledge graph completion

**DOI:** 10.1038/s41598-025-18260-y

**Published:** 2026-06-12

**Authors:** Panfeng Chen, Minggan Xiao, Xiuning Wang, Hui Li, Xibin Wang, Xin Zhou

**Affiliations:** 1 Guizhou Provincial Laboratory of Big Data, Guizhou University, Guiyang, Guizhou China; 2 Department of Computer Science and Technology, Guizhou University, Guiyang, Guizhou China; 3 Guizhou Institute of Technology College of Big Data, Guiyang, Guizhou China

**Keywords:** Knowledge graph completion, Knowledge embedding, Translation-based model, Linear transformation, Translation equation, Information technology, Scientific data, Applied mathematics

## Abstract

The pessimistic conclusions from previous research on the *Expressive Power* of translating approaches for knowledge graph completion are investigated and rethought. To this end, a novel model *RosE* is formulated by introducing two degrees of freedom and outperforms traditional translation-based models on widely used datasets such as FB15k, WN18, FB15k237, and WN18RR. Every new freedom is a vector in the model, the operation of which multiplies with entity and relation embeddings, rotating them to a new position. Consequently, the head entity and relation embedding are equal to the tail entity. Fortunately, the intrinsic limitations merely exist in this research line when the model is trained in real vector space, not in other spaces such as trigonometric functions and complex. The experimental and theoretical results, together with the newly proposed model RosE, also confirm this conclusion. Therefore, the findings in this work do not discourage further exploration in this research line, but rather avoid those with discouraging outcomes. In short, this paper clarifies that the limitations of the translation approach for knowledge graph completion are specific conditions that only involve partial models. That is, the research line of translation approach is still promising when certain known pitfalls are avoided.

## Introduction

**Fig. 1:**
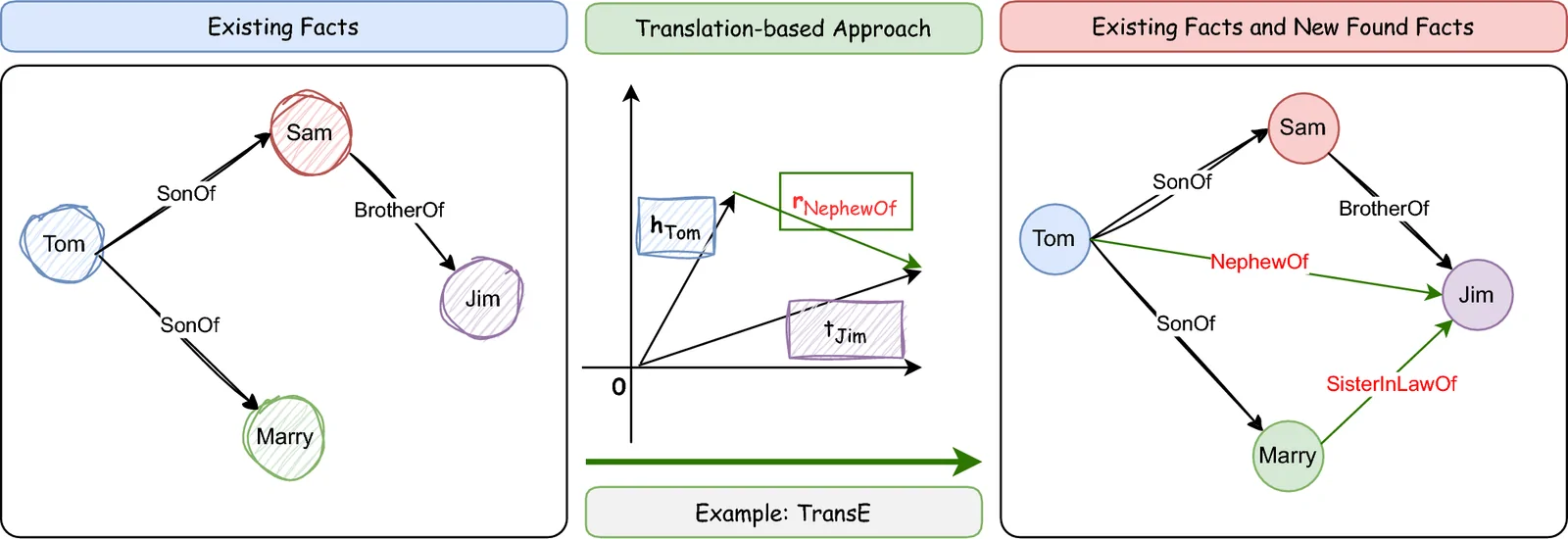
An example illustrates the knowledge graph completion. The relations Nephewof and SisterInLawOf are inferred from the known facts.

Knowledge graphs (KGs) are broadly applied to information retrieval^1,2^, healthcare^3^, question answering^4,5^, and even large language model (LLM) enhancement^6^ to reduce the hallucination. As a result, they are the key components of a smart system. Unfortunately, they are typically incomplete^7,8^ and heavily impacting the downstream works. To address the issue, there is massive work on the knowledge graph completion (as shown in Fig. 1) from both industry and academic fields.

There are dozens of models proposed for this end. The translation-based research line is one of the strong branches of knowledge graph completion. In this research line, the knowledge graph completion (KGC) task is a binary classification. The key point is to determine whether a specific relation exists between an entity pair. The typical model TransE^9^. Its extensions such as TransH^10^, TransD^11^, TransR^12^, STransE^13^, FTransE^14^, as shown in Table 1 are attributed to translating approach, which is based on the approximate equation $\mathbf{h}+\mathbf{r}\approx \mathbf{t}$ and it is termed *translation equation* for fluently writing. Nevertheless, the research in^15^ concludes that *FSTransE and its variants of translating approaches mentioned above have the restrictions pointed out by Proposition 2* (refer to A). This conclusion means that the intrinsic deficits of this kind models limit the expressive power. It hurts the positive attitude towards the expressive power of models from this research line. The models, such as STransE and TransR, are devoted to bolstering their expressive power. The proposed model FSTransE in the work^15^ is considered to be a general form of them by increasing one degree of freedom. However, the prospects for FSTransE and its counterparts seem murky.

Recent models, such as RotatE^16^, RatE^17^, TimE^18^, and TransO^19^, have demonstrated the potential of the translating approach for knowledge graph completion, challenging the pessimistic conclusions from the paper^15^ about its limitations. Unfortunately, there is no further research on this topic, and works in this line are becoming increasingly scarce. To better understand the expressive power of the translating approach with reasonable time complexity, a general and straightforward model *RosE* is proposed, which Rotates and scales Embeddings to help demonstrate this idea. In this model, two degrees of freedom are used, making it more general than FSTransE. The theoretical and empirical results in this work reveal the intrinsic restrictions of these models and show that RosE achieves state-of-the-art performance within this research line. Moreover, the proposed model confirms that the limitations of the translating approach are not always valid, but depend on certain conditions.

### Contributions

On this basis of those mentioned above, this work has three main insights or contributions as follows:

(i) The models are linearly transformed from the translation equation, which cannot overcome its inherent limitations, but non-linearly transformed ones can.
(ii) The expressive power of the translation-based research is still as strong as any other research line, which is still full of chance in this research line.
(iii) The proposed model, RosE, enhances the expressive power by introducing two degrees of freedom and achieves state-of-the-art performance in its research line at low cost.

## Backgraound and related works

In this section, the important notations and concepts in this work are briefly introduced.

### Notations and basic knowledge

A KG can be represented by a tuple $\mathcal {G} = (\mathcal {E},\mathcal {R})$, where $\mathcal {E},\mathcal {R}$ are the sets of entities and relations, respectively. A KG consists of triples in the form of (*h*, *r*, *t*), where $(h,r,t)\in \mathcal {G}$, $h,t\in \mathcal {E}$ and $r\in \mathcal {R}$. Let $\mathcal {F}$ be the set of all possible triples in the world, i.e., $\mathcal {F} = \mathcal {E}\times \mathcal {R}\times \mathcal {E}$, and $\mathcal {G}\subseteq \mathcal {F}$ since KGs are typically incomplete. Let *Δ* be the set of positive samples in KG (i.e., $\Delta \subseteq \mathcal {G}$) and *Δ '* be a set of negative ones (i.e., $\Delta '\nsubseteq \mathcal {G}$). The triple to be completed is usually in the form of (*h*, *r*, ?) or $(?,r,t)\in \mathcal {F}$ (Knowledge graph completion usually predicts between two existing entities under the closed-world assumption.). It is known that an embedding is from a projection by a function $f:v\rightarrow \mathbf{v}\in \mathbb {R}^d$ that maps any element of a fact (i.e., entity or relation) to a vector $\mathbf{v}$. The embeddings of head entity, relation, and tail entity are denoted as $\mathbf{h}$, $\mathbf{r}$, and $\mathbf{t}$ ($\mathbf{h}, \mathbf{t}, \mathbf{r} \in \mathbb {R}^{d}$), respectively. When head and tail entities do not need to be distinguished, they are denoted as a common bold letter $\mathbf{e}$ to simplify the notation. Furthermore, *d* is used when the dimensions of entity and relation vectors are equal; otherwise, they are denoted as $d_e$ and $d_r$. $N_e$, $N_r$ and $N_t$ are the numbers of entities, relations and triples in KG, respectively. Embedding-based KGC is an approach for link prediction that learns the embeddings in a vector space. In the following sections, $\mathbf{0}^d$ and $\mathbf{1}^d$ are *d*-dimensional vectors whose all elements are 0 s and 1 s, respectively.

In KGs, some unobserved relations may exist between entity pairs and have underlying features that can be mined. A relation *r* is *reflexive* on a set of entities $\mathcal {E}$ if $(e,r,e)\in \mathcal {F}$ for all $e\in \mathcal {E}$. A relation *r* is *symmetric* on a set of entities $\mathcal {E}$ if $(h,r,t)\in \Delta \Longleftrightarrow (t,r,h)\in \Delta$. A relation *r* is *transitive* on a set of entities $\mathcal {E}$ if $(h,r,t_1)\in \Delta \wedge (t_1,r,t)\in \Delta \Rightarrow (h,r,t)\in \Delta$ for all $h,t_1,t\in \mathcal {E}$.

In the following sections, we use the operator *⋆*: $\mathbb {R}^d\star \mathbb {R}^d\rightarrow \mathbb {R}^d$ to denote element-wise division of vectors, i.e., $[\mathbf{b}]_i\star [\mathbf{a}]_i = [\frac{\mathbf{b}}{\mathbf{a}}]_i = \frac{[\mathbf{b}] _i}{[\mathbf{a}]_i}$, and $b \star [\mathbf{a}]_i = [\frac{b}{ \mathbf{a}}]_i = \frac{b}{[\mathbf{a}]_i}$. We also use the operator *∘*: $\mathbb {R}^d \circ \mathbb {R}^d \rightarrow \mathbb {R}^ d$ to denote Hadamard product of vectors, i.e., $[\mathbf{a} \circ \mathbf{b}]_i = [a]_i \cdot [b]_i$. The result of both operators is still a *d*-dimensional vector.

### Translation equation

**Fig. 2:**
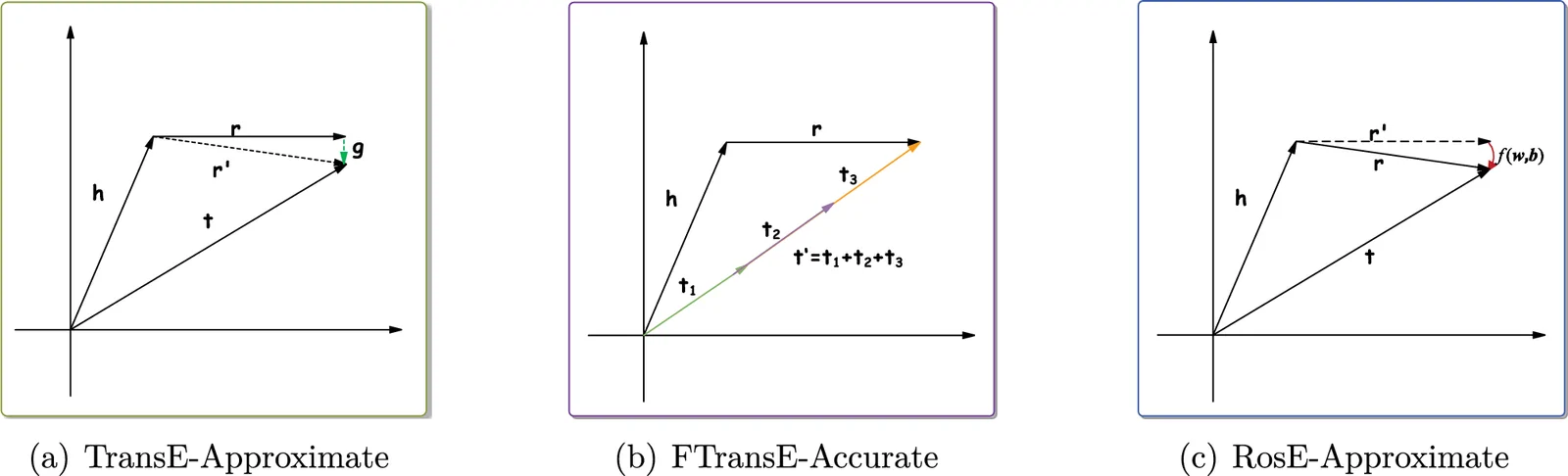
Comparison among the proposed model RosE and FTransE, TransE.

Translation-based models are a type of embedding-based models that stem from the experimental results of the famous natural language processing approach Word2Vec^20^. In the experiments, there exist quantifying relations between word embeddings such as $\mathbf{v}_{Berlin} - \mathbf{v}_{Germany} \approx \mathbf{v}_{Paris} - \mathbf{v}_{France}$. This implies that Berlin is the *capital of* Germany and Paris is the *capital of* France, which corresponds to the approximately equal differences between their word vectors. The geometric meanings between word embeddings are illustrated in Fig. 2a and c .

As we know, a fact in KG is typically in the form of words, which can be denoted as word embeddings. Therefore, we have an equation that follows the assumption of additive compositionality^21^, and its mathematical form is as follows:

1 $$\begin{aligned} \mathbf{h} + \mathbf{r} \approx \mathbf{t}, \end{aligned}$$

where $\mathbf{h}$, $\mathbf{r}$ and $\mathbf{t}$ are the embeddings trained on known facts, respectively. As mentioned earlier, Eq. (1) is called the *translation equation* in this work. Its variants include forms such as $\mathbf{h} \approx \mathbf{t} - \mathbf{r}$ or $\mathbf{t} - \mathbf{h} \approx \mathbf{r}$. All of these forms are equivalent to Eq. (1).

Apparently, the form of the equation is simple and effective. It is so simple as not expressive enough for complicated relations, as pointed out in work^10^. Moreover, this equation is an approximation, as illustrated in Fig. 2a . There is a gap between the vectors ($\mathbf{h} + \mathbf{r}$) and $\mathbf{t}$, meaning that there is no absolute equivalence as demonstrated in Fig. 2b (i.e., FTransE) and c (i.e., RosE). The relation embedding $\mathbf{r}'$ is the expected one, and $\mathbf{r}$ is the result of $(\mathbf{t} - \mathbf{h})$ (i.e., the translation of entity embeddings). Therefore, the gap $\mathbf{g}$ is the difference between these two vectors, namely, $\mathbf{g} =\mathbf {r'} - \mathbf{r}$ and $\mathbf{g}\ne \mathbf{0}$ (as shown in Appendix B and Fig. 2b). It is potentially possible to address this problem by rotating the vector to exactly equal. As a result, the model is more expressive.

To retain the advantages of TransE and improve its performance, the proposed model increases the degree of freedom by rotating and scaling the embeddings, thereby solving this problem.

The proposed model is a translation-based one. As a consequence, the model TransE and its extensions are closely related to it. These models fall into two categories, according to the manner in which the translation equation works with them, namely, linear or non-linear. In this paper, we focus on the former.

### Translation-based model

The translation-based approach is a crucial research line for the KGC task, also known as the translating model. They mainly include the model TransE^21^ and its variants, which are also referred to as extensions. The models from this research line are efficient and straightforward. The key component of the model is the scoring function, which serves as a criterion for scoring link prediction, as defined in Eq. (1).

The proposed model is a translation-based one, and as a consequence, the TransE model and its extensions are closely related to it. These models fall into two categories based on how the translation equation works, namely, linear or non-linear. In this paper, we focus on the former.

The translation-based approach is a crucial research area for the KGC task, also known as the translating model. It primarily includes the TransE model^21^ and its variants, also known as extensions. The models from this research line are efficient and straightforward. The key component of the model is the scoring function, which serves as a criterion for scoring link predictions according to Eq. (1).

#### Representation

Translation-based models project KG data into a vector space as words. Each literal element, such as an entity or relation, is a symbol mapped to a vector. These vectors are low-dimensional and distributed, making them easily computable by machine or deep learning algorithms. The additive compositionality assumption is the key principle governing this type of model. The initial vectors are typically generated from a specific distribution, such as a uniform or Bernoulli distribution. To enhance the model’s expressive power, relations can also be represented as matrices, such as in TransD^11^, TransR^12^, etc. This way, the model can capture more relational features. In the proposed model RosE, entities and relations are projected into a *d*-dimensional real vector space. On the other hand, in the new models RotatE^16^, RatE^17^, and TimE^18^, their entities and relations are projected into a complex vector space, and the translation equation works in an exponential vector space.

#### Scoring function

In this section, representative translation-based models are reviewed as illustrated in Table 1.

**Table 1:** The typical models transformed from translation equation.

Model	Scoring Function	Parameters	Transform	Complexity
TransE^21^	$\vert \mathbf{h}+\mathbf{r}-\mathbf{t}\vert ^l$	$\mathbf{h},\mathbf{r},\mathbf{t}\in \mathbb {R}^d$	linear	$\mathcal {O}(N_t d)$
TransH^10^	$\vert \mathbf{h}_{\bot }+\mathbf{r}-\mathbf{t}_{\bot }\vert ^l$, where $\mathbf{h}_{\bot } = \mathbf{h}-\mathbf{w}_{r}^{\top }\mathbf{h}$ and $\mathbf{t}_{\bot } = \mathbf{t}-\mathbf{w}_{r}^{\top }\mathbf{t}$.	$\mathbf{h},\mathbf{r},\mathbf{t},\mathbf{w}_r\in \mathbb {R}^d$ and $\Vert \mathbf{w}_r\Vert ^2 = 1$.	linear	$\mathcal {O}(N_t d^2)$
TransR^12^	$\vert \mathbf{h}_{r}+\mathbf{r}-\mathbf{t}_{r}\vert ^l$, where $\mathbf{h}_{r}=\mathbf{h}\mathbf{M}_{r}$ and $\mathbf{t}_{r}=\mathbf{t}\mathbf{M}_{r}$.	$\mathbf{h},\mathbf{r},\mathbf{t}\in \mathbb {R}^d$ and $\mathbf{M}_{r}\in \mathbb {R}^{N_r\times d}$.	linear	$\mathcal {O}(N_t N_r d_r)$
TransD^11^	$\vert \mathbf{h}_{\bot }+\mathbf{r}-\mathbf{t}_{\bot }\vert ^l$, where $\mathbf{h}_{\bot } = \mathbf{M}_{rh}\mathbf{h}$, $\mathbf{t}_{\bot } = \mathbf{M}_{rt}\mathbf{t}$, $\mathbf{M}_{rh} = \mathbf{r}_{p}\mathbf{h}_{p}^{\top } + \mathbf{I}$ and $\mathbf{M}_{rt} = \mathbf{r}_{p}\mathbf{t}_{p}^{\top } + \mathbf{I}$.	l$\mathbf{h},\mathbf{r},\mathbf{t}\in \mathbb {R}^d$, $\mathbf{M}_{rh}\in \mathbb {R}^{d_r\times d_e}$, $\mathbf{M}_{rh}\in \mathbb {R}^{d_r\times d_e}$ and $\mathbf{I}\in \mathbb {R}^{d_r\times d_e}$.	linear	$\mathcal {O}(N_t d_e d_r)$
STransE^13^	$\vert \mathbf{h}_{r}+\mathbf{r}-\mathbf{t}_{r}\vert ^l$, where $\mathbf{h}_{r}=\mathbf{h}\mathbf{M}_{r}$ and $\mathbf{t}_{r}=\mathbf{t}\mathbf{M}_{r}$.	$\mathbf{h},\mathbf{r},\mathbf{t}\in \mathbb {R}^d$ and $\mathbf{M}_{r}\in \mathbb {R}^{d\times d}$.	linear	$\mathcal {O}(N_t d^2)$
GTrans^22^	$\vert \mathbf{h}+\mathbf{r}-\mathbf{t}\vert ^l$, where $\mathbf{h}=\alpha \mathbf{h}_e + \beta \mathbf{h}_a^{\top }\mathbf{h}_e\mathbf{r}_a$ and $\mathbf{t}=\alpha \mathbf{t}_e + \beta \mathbf{t}_a^{\top }\mathbf{t}_e\mathbf{r}_a$.	$\mathbf{h},\mathbf{r},\mathbf{t},\mathbf{h}_a,\mathbf{t}_a,\mathbf{h}_e,\mathbf{t}_e,\mathbf{r}_a\in \mathbb {R}^d$ and $\alpha ,\beta \in \mathbb {R}$.	linear	$\mathcal {O}(N_t d^3)$
RotatE^16^	$\vert \mathbf{h}\circ \mathbf{r}-\mathbf{t}\vert ^l$	$\mathbf{h},\mathbf{r},\mathbf{t}\in \mathbb {C}^d$ and $\forall i:\vert \mathbf{r}\vert _i$ = 1.	non-linear	$\mathcal {O}(N_t d)$
RatE^17^	$\vert \mathbf{h}\odot _\mathbf {W^{(r)}}\mathbf{r}-\mathbf{t}\vert ^l$	$\mathbf{h},\mathbf{r},\mathbf{t}\in \mathbb {C}^d$, $\forall i:\vert \mathbf{r}\vert _i$ = 1 and $\mathbf{W}^{(r)}\in \mathbb {R}^{d_e\times d_r}$.	non-linear	$\mathcal {O}(N_t d_e d_r)$
TimE^18^	$\vert \mathbf{h}+\mathbf{r}-\mathbf{t}_{\bot }\vert ^l + \mathbf{h}+\mathbf{r}-\mathbf{t}_{\bot }\vert ^l$, where $\mathbf{t}_{\bot }=\mathbf{t}^{\top } diag(\cos \boldsymbol{\theta }^t_r)$ and $\mathbf{h}_{\bot }=\mathbf{h}^{\top } diag(\cos \boldsymbol{\theta }^t_r)$.	$\mathbf{h},\mathbf{r},\mathbf{t}\in \mathbb {R}^d$,$\cos \boldsymbol{\theta }^h_r\in [0,2\pi )^d$ and $\cos \boldsymbol{\theta }^h_r\in [0,2\pi )^d$.	non-linear	$\mathcal {O}(N_t d_e d_r)$

In KGs, there are mainly two kinds of prediction (*h*, *r*, ?) and (?, *r*, *t*) under the closed-world assumption. In other words, the KGC task is usually to predict the existence of a new fact by checking how well the entity and relation embeddings satisfy the translation equation. To complete a triple, the missing entity can be predicted by computing and ranking the scores of candidate entities. In this case, all entities (except itself) are potential candidates. They are ranked according to their scores, and a better rank order means more compliance with the criterion rule. TransE is the canonical model in translation-based models, and others are extended from it; therefore, the paradigm of the rank criterion is similar to it. Its scoring function is defined as

2 $$\begin{aligned} f_{TransE}(h,r,t) = \vert \mathbf{h} + \mathbf{r} -\mathbf{t}\vert ^{l}, \end{aligned}$$

where $\mathbf{h},\mathbf{r}\text { and }\mathbf{t}$ are the embeddings trained by the observed facts in KGs and *l* ($l\in {\ell _{1},\ell _{2}}$) is the vector or matrix norm. To overcome the disadvantage of TransE and improve the expressive power of the model for reflexive, one-to-many, many-to-one, and many-to-many relations, one can project them onto a hyperplane to distinguish them from each other. The scoring function of this model is defined as

3 $$\begin{aligned} f_{TransH}(h,r,t) = \vert \mathbf{h}_{\bot } + \mathbf{d}_r -\mathbf{t}_{\bot }\vert ^{l}, \end{aligned}$$

where $\mathbf{h}_{\bot } = \mathbf{h}-\mathbf{w}_{r}^{\top }\mathbf{h}\mathbf{w}_{r}$ and $\mathbf{t}_{\bot } = \mathbf{t}-\mathbf{w}{r}^{\top }\mathbf{t}\mathbf{w}{r}$ are the projections of $\mathbf{h}$ and $\mathbf{t}$ on the hyperplane. And $\mathbf{d}_{r}$ is the projection of $\mathbf{r}$ on the hyperplane $\mathbf{w}_{r} \in \mathbb {R}^d$. In the paper^12^, it is noted that these models project the triples into the same semantic space, which is insufficient to reflect real facts accurately. TransR is proposed to overcome this deficit. The scoring function is defined as

4 $$\begin{aligned} f_{TransR}(h,r,t) = \vert \mathbf{h}_{r} + \mathbf{r} -\mathbf{t}_{r}\vert ^{l}, \end{aligned}$$

where $\mathbf{h}_{r}=\mathbf{h}\mathbf{M}_{r}$ and $\mathbf{t}_{r}=\mathbf{t}\mathbf{M}_{r}$ are the projections of $\mathbf{h}$ and $\mathbf{t}$. $\mathbf{M}_{r}\in \mathbb {R}^{N_r\times d}$ is a matrix concatenated by the relation vectors. Afterwards, an extended version named TransD^11^ is proposed to improve the performance and reduce the complexity of TransR/CTransR by factorizing this matrix into two vectors in a fine-grained manner. As a result, the scoring function is transformed as follows.

5 $$\begin{aligned} f_{TransD}(h,r,t) = \vert \mathbf{h}_{\bot } + \mathbf{r} -\mathbf{t}_{\bot }\vert ^{l}, \end{aligned}$$

where $\mathbf{h}_{\bot } = \mathbf{M}_{rh}\mathbf{h}$, $\mathbf{t}_{\bot } = \mathbf{M}_{rt}\mathbf{t}$. $\mathbf{M}_{rh}\in \mathbb {R}^{d_r\times d_e}$ and $\mathbf{M}_{rh}\in \mathbb {R}^{d_r\times d_e}$ are matrices mapped from head and tail entities, respectively. Furthermore, $\mathbf{M}_{rh} = \mathbf{r}_{p}\mathbf{h}_{p}^{\top } + \mathbf{I}$ and $\mathbf{M}_{rt} = \mathbf{r}_{p}\mathbf{t}_{p}^{\top } + \mathbf{I}$, $\mathbf{I}\in \mathbb {R}^{d_r\times d_e}$ is an identity matrix and $\mathbf{r}_p\in \mathbb {R}^{d_r}$, $\mathbf{h}_p\in \mathbb {R}^{d_e}$ and $\mathbf{t}_p\in \mathbb {R}^{d_e}$ are the factor vectors factorized from matrices $(\mathbf{M}_{rh} - \mathbf{I})$ and $(\mathbf{M}_{rh} - \mathbf{I})$, respectively. The entity and relation have different dimensions. Moreover, in the scoring function of extended models such as TransH, TransR, and TransD, the norm *l* is $\ell _{2}$.

Translation-based models are a popular research direction for knowledge graph embedding. Some recent models, such as RotatE^16^ and RatE^17^, embed entities and relations in a complex vector space and use non-linear operations to capture the semantic interactions. Another model, TimE^18^, can be viewed as a modulus variant of RotatE, which introduces a trigonometric function to model periodic relations. These models demonstrate that translation-based models can achieve promising results with non-linear transformations.

However, some translation-based models also introduce additional assumptions or complexities that may limit their scalability and efficiency. For example, GTrans^22^ assumes that the samples in knowledge graphs follow a Gaussian distribution and uses a probabilistic framework to model uncertainty. Alternatively, some models resort to neural networks to enhance the expressive power of translation-based models, such as NTransGH^23^ and TransGate^24^. These models are more complicated and less efficient than simple translation-based models. Similar challenges also exist in other research paradigms such as factorization-based, logical rules-based, and neural networks-based models.

Factorization-based models, such as RESCAL^25^, DistMult^26^, ComplEx^27^, TuckER^28^, have full expressiveness^15^. However, they still rely on neural networks to improve their performance^29,30^. Logical rules-based models, which were originally based on symbolic logic and had limited generalization ability, have also adopted neural networks in recent works, such as MLN^31^, Neural LP^32^, DRUM^33^, MINERVA^34^, RNNLogic^35^. These models achieve promising results but at the cost of high complexity and computational expense. This contradicts the practical needs of applications. Therefore, we focus on translation-based models that are simple and efficient.

#### Loss function

The loss function is a crucial component that guides the model toward converging on the optimal solution. For translation-based models, they usually adopt a similar form of loss function to TransE. It can be expressed as follows.

6 $$\begin{aligned} \mathcal {L}_{TransE} = \sum _{(h,r,t)\in \Delta }\sum _{(h',r,t')\in \Delta '}\max \{0, \gamma + f_{TransE} - f^{'}_{TransE}\}, \end{aligned}$$

where the function $\max \{0,x\}$ means the value of it is no less than 0, and the set of negative samples is

7 $$\begin{aligned} \Delta ' = \{(h',r,t)\vert h'\in \mathcal {E}\}\cup \{(h,r,t')\vert t'\in \mathcal {E}\}. \end{aligned}$$

Typically, this is also their objective function, as it lacks a regularization term.

#### Training and optimization

Most of the models discussed above use a loss function without a regularization term as their training objective. For example, TransE aims to minimize the loss function $\mathcal {L}_{TransE}$. This is a non-convex function, so Stochastic Gradient Descent (SGD) is applied for optimization. However, this may lead to a local optimum. Moreover, many loss functions of this kind do not include regularization terms.

## Results

To evaluate the performance of the proposed model empirically, several experiments have been devised. The details are presented in this section.

### Datasets

To evaluate the proposed model, six widely used datasets have been selected. These datasets can be divided into three groups. The first group includes the Kinships^36^ and UMLS^37^ datasets, which are small yet widely used for evaluating KGC models. The former is not a real KG but a set of relationships among kin in a tribe, while UMLS is a real-world KG. The second group consists of large-scale datasets FB15K and WN18, which are used to evaluate the scalability of models and are very popular for KGC model evaluation. The third group includes FB15K237 and WN18RR, which are considered more challenging and closer to real-world KGs. They have addressed the test set leakage problem^38,39^ and are becoming increasingly popular for evaluating new KGC models. The statistics for these datasets are shown in Table 2.

**Table 2:** The datasets used in the experiments.

Dataset	$N_e$	$N_r$	$N_t$
Kinships	104	25	10,686
UMLS	135	46	6,529
FB15K	14,951	1,345	483,142
WN18	40,943	18	141,442
FB15K237	14,541	237	272,115
WN18RR	40,943	11	86,835

### Experimental setup

The proposed model was implemented using Python 3.7.9 and executed on a computer equipped with an Intel Core i7-7700 CPU, featuring eight cores and a base frequency of 3.60 GHz, along with 8 GB of RAM. The optimization algorithm used was SGD with the Adam method. The source code is available on GitHub at the following site: https://github.com/gzupanda/RosE.

#### Hyperparameters

The hyperparameters mainly include the coefficients of weights and biases, as well as the optimization algorithm, SGD. The coefficient of correction terms is simple, and the model performs well only when $\delta _{1}=\delta _{2}=0.5$ holds, and they are not listed in Table 3. In other words, the two parameters are set to 0.5 during the training process. The left hyperparameters are searched in a grid manner. And their values for being searched as follows: $d\in \{50,100,150,200,300\}$, $\mu \in \{0.1,0.2,0.5,1,2,3,4,5\}$, $\gamma \in \{0.01,0.02,0.05,0.1,0.2,0.5\}$, and four more learnable hyperparameters for the proposed model RosE are $\mu _A (\mu _B) \in \{0.1,0.2,0.5,1,2\}$ and $\lambda _A (\lambda _B)\in \{0.00.1, 0.002, 0.005, 0.01, 0.02, 0.05, 0.1, 0.2, 0.5, 1\}$, where *d* is embedding dimension, *μ* is learning rate, *γ* is the margin in SGD, $\mu _A (\mu _B)$ is the coefficient of weight and $\lambda _A (\lambda _B)$ is the coefficient of bias in regularization term. Table 3 recorded the parameters when the model achieves the best performance results.

**Table 3:** Hyperparameters for the best performance on the corresponding datasets of both versions of the proposed model RosE.

Dataset	Parameters								
	$V_{Model}$	*d*	*b*	*μ*	*γ*	$\mu _{A}$	$\mu _{B}$	$\lambda _{A}$	$\lambda _{B}$
Kinships	RosE$_{1l}$	200	427	5	0.2	0.5	0.5	0.001	0.001
	RosE$_{2l}$	200	427	5	0.1	0.5	0.5	0.001	0.004
UMLS	RosE$_{1l}$	200	522	1	0.2	0.5	0.5	1	1
	RosE$_{2l}$	150	522	2	0.03	1	0.5	0.04	0.02
FB15k	RosE$_{1l}$	300	4831	0.5	0.5	0.5	0.5	0.1	0.5
	RosE$_{2l}$	300	4831	0.5	0.02	0.5	0.5	0.5	0.5
WN18	RosE$_{1l}$	200	1414	3	0.05	1	0.5	0.01	0.04
	RosE$_{2l}$	200	141	2	0.02	0.5	0.5	0.5	0.5
FB15k237	RosE$_{1l}$	300	2721	2	0.03	1	0.5	0.04	0.02
	RosE$_{2l}$	300	2721	2	0.03	0.5	0.5	0.004	0.002
WN18RR	RosE$_{1l}$	50	868	5	0.03	1	0.5	0.04	0.02
	RosE$_{2l}$	200	868	3	0.03	0.5	0.5	0.04	0.02

#### Evaluation protocol

In this paper, the results are reported using the metrics MR (Mean Rank), MRR (Mean Reciprocal Rank), and Hits@N ($N\in {1,3,10}$). Let $r_h^i$ and $r_t^i$ denote the rank of the head and tail entities in the *i*th triple from the testing set $\mathcal {T}$ and let $\vert \mathcal {T}\vert$ represent the number of triples in the testing set. Let $N_h^i$ and $N_t^i$ be the number of head and tail entities within the top N for the *i*th triple in testing set $\mathcal {T}$. For the head entity of the *(i+1)*th triple, if $N_h^i\le N$, then $N_h^{(i+1)} = N_h^i + 1$, otherwise $N_h^{(i+1)} = N_h^i + 0$. The situation is similar for tail entities. Therefore, these metrics are computed as follows.

**Mean Rank** refers to the average rank of the predicted entity among all candidate entities in the testing set. This metric is reported to facilitate comparison with other models, as most translation-based models also report it. It is defined as follows:

$$\begin{aligned} \text {MR} = \frac{\sum _{i=1}^{\vert \mathcal {T}\vert }(r_h^i + r_t^i)}{2\vert \mathcal {T}\vert }. \end{aligned}$$

**Mean Reciprocal Rank** represents the average reciprocal rank of candidate entities, including both head and tail entities. It is defined as follows:

$$\begin{aligned} \text {MRR}= \frac{\sum _{i = 1}^{\vert \mathcal {T}\vert }(\frac{1}{r_h^i} + \frac{1}{r_t^i})}{2\vert \mathcal {T}\vert }. \end{aligned}$$

**Hits@N** represents the average percentage of head and tail entities in the testing set that rank within the top N. It is defined as follows:

$$\begin{aligned} \text {Hits@N} = \frac{\sum _{i = 1}^{\vert \mathcal {T}\vert } (N_h^i + N_t^i)}{2\vert \mathcal {T}\vert }\times 100\%. \end{aligned}$$

For the MR metric, lower values are preferable, while for MRR and Hits@N, higher values are more desirable. Notably, since a significant number of translation-based models report the MR and Hits@10 metrics for the FB15k and WN18 datasets, the experimental results also follow this paradigm. For the other four datasets, MRR, Hits@1, Hits@3 and Hits@10 (i.e., $N\in {1,3,10}$) are reported for ease of comparison.

In the proposed model, each score is computed using the scoring function for a candidate entity based on known entity and relation embeddings. The scores for candidate entities are then ranked in ascending order. For the rank of the golden entity: if it does not rank first, is it in the top 3? Or in the top 10? The proportion of these ranks among all test set entities is measured by the metrics Hits@1, Hits@3, and Hits@10. Since the golden entity is selected by rank, ranking-related metrics such as MR and MRR are also used for KGC. To achieve optimal metric results locally or globally, SGD is used to optimize the model since it is non-convex.

### Results and analysis

This section contains three tables that record the performance results for the three groups of widely used datasets. Analyses corresponding to each table are provided. In these tables, *Filt.* stands for Filtered, meaning that if an expected negative sample is a positive one in the KG, it is considered a false negative sample and can lead to low model performance. Such samples must be filtered out to avoid this problem. *Strategy* refers to the method used for generating negative samples, which typically follow either a uniform^21^ or Bernoulli^10^ distribution. *unif.* indicates that negative sampling follows a uniform distribution, which is the most commonly used strategy, while *bern.* suggests that it follows a Bernoulli distribution where negative samples are also produced by corrupting and replacing entities. The best performance results are shown in bold, while secondary results are italic.

#### Performance on small datasets

This group of datasets is relatively small compared to others. However, they also typically use datasets for evaluating the new proposed models, especially in the factorization-based, logical rules-based, and even neural networks-based approaches.

**Table 4:** The results for small datasets are shown.

Model	Kinships				UMLS			
	MRR	Hits@			MRR	Hits@10		
	Filt.	1	3	10	Filt.	1	3	10
TransE$^{\clubsuit }$	0.326	2.4	54.8	88.3	0.639	34.0	93.1	98.4
CP$^{\clubsuit }$	0.759	63.1	86.7	97.4	0.858	*75.9*	94.8	98.7
RESCAL$^{\clubsuit }$	**0.801**	**68.9**	**90.1**	*97.6*	*0.854*	74.1	96.0	*99.2*
DistMult$^{\clubsuit }$	0.492	31.8	57.8	89.1	0.662	53.0	75.8	90.5
ComplEx$^{\clubsuit }$	0.737	59.3	85.9	**98.2**	**0.896**	**82.9**	95.6	99.0
ComplEx-N3$^{\spadesuit }$	0.605	43.7	71.0	92.1	0.791	68.9	87.3	95.7
TuckER$^{\spadesuit }$	0.603	46.2	69.8	86.3	0.732	62.5	81.2	90.9
RotatE$^{\spadesuit }$	0.651	50.4	75.5	93.2	0.744	63.6	82.2	93.9
MLN$^{\spadesuit }$	0.351	18.9	40.8	70.7	0.688	58.7	75.5	86.9
Boosted RDN$^{\spadesuit }$	0.469	39.5	52.0	56.7	0.227	14.7	25.6	37.6
PathRank$^{\spadesuit }$	0.369	27.2	41.6	67.3	0.197	14.8	21.4	25.2
Neural LP$^{\spadesuit }$	0.302	16.7	33.9	59.6	0.483	33.2	56.3	77.5
DRUM$^{\spadesuit }$	0.334	18.3	37.8	67.5	0.548	35.8	69.9	85.4
MINERVA$^{\spadesuit }$	0.401	23.5	46.7	76.6	0.564	42.6	65.8	81.4
CTP$^{\spadesuit }$	0.335	17.7	37.6	70.3	0.404	28.8	43.0	67.4
RNNLogic$^{\spadesuit }$	0.639	49.5	73.1	92.4	0.745	63.0	83.3	92.4
RNNLogic1$^{\spadesuit }$	0.722	59.8	81.4	94.9	0.842	77.2	89.1	96.5
RulE^40^	0.736	61.5	82.4	95.7	0.867	79.7	92.5	97.2
RosE$_{1l}$(ours)	0.432	17.6	62.0	93.6	0.833	68.8	**97.9**	**99.6**
RosE$_{2l}$(ours)	*0.766*	*63.7*	*87.9*	*97.6*	0.801	63.6	*96.4*	99.4

The [$^{\clubsuit }$] symbol indicates performance results obtained by running the code provided in the paper^27^, while the [$^{\spadesuit }$] symbol indicates results reported in the paper^35^.

Table 4 shows that the two versions of the proposed model, RosE, achieved SOTA or competitive performance results on small datasets. These datasets include translation-based models such as TransE, as well as factorization-based, logical rules-based, and neural networks-based models. On the UMLS dataset, RosE achieved SOTA performance over all three types of models despite RESCAL^25^ being proven to be fully expressive^15^. The proposed model significantly outperformed translation-based models. For example, RosE$_{1l}$ outperformed TransE by 30.36% in MRR, 102.35% in Hits@1, 5.16% in Hits@3 and 1.22% in Hits@10. On the Kinships dataset, RosE outperformed TransE by a large margin and even performed better than RotatE^16^, which was more general than TransE in all reported metrics (17.67% in MRR, 26.38% in Hits@1, 16.42% in Hits@3 and 4.72% in Hits@10). The results for other models were also competitive. This experiment demonstrates that rotating and scaling embeddings with weight and bias vectors can improve model performance by bridging the gap between real and expected relations.

#### Performance on large datasets

These two datasets serve as benchmark standards, which were first introduced to evaluate the performance of the TransE model^21^. Therefore, lots of translation-based models are evaluated on them, though they are considered to be test linkage^38,39^.

**Table 5:** The link prediction performance results on larger datasets FB15k and WN18.

Model	Str.	FB15K				WN18			
		MR		Hits@10		MR		Hits@10	
		Filt.	Raw	Filt.	Raw	Filt.	Raw	Filt.	Raw
TransE$^{\clubsuit }$	unif.	125	243	47.1	34.9	251	263	89.2	75.4
TransH$^{\clubsuit }$	unif.	84	211	58.5	42.5	303	318	86.7	75.4
TransH$^{\clubsuit }$	bern.	87	212	64.4	45.7	388	401	82.3	73.0
TransR$^{\clubsuit }$	unif.	78	226	65.5	43.8	219	232	91.7	78.3
TransR$^{\clubsuit }$	bern.	77	198	68.7	48.2	225	238	92.0	79.8
CTransR$^{\clubsuit }$	unif.	82	233	66.3	44.0	230	243	92.3	78.9
CTransR$^{\clubsuit }$	bern.	75	199	70.2	48.4	218	231	92.3	79.4
TransD$^{\clubsuit }$	unif.	67	211	74.2	49.4	229	242	92.5	79.2
TransD$^{\clubsuit }$	bern.	91	194	77.3	53.4	212	224	92.2	79.6
STransE$^{\clubsuit }$	unif.	69	220	78.4	51.5	211	224	93.2	80.8
STransE$^{\clubsuit }$	bern.	68	219	79.7	51.6	206	219	93.4	80.9
TransPES^41^	unif.	66	198	67.3	48.1	212	223	81.3	71.6
TransE-RS$^{\clubsuit }$	unif.	**62**	**161**	72.3	53.1	348	362	93.7	80.3
TransE-RS$^{\clubsuit }$	bern.	*63*	**161**	72.1	53.2	371	385	93.7	80.4
TransH-RS$^{\clubsuit }$	unif.	64	*163*	72.6	*53.4*	389	401	*94.7*	*81.2*
TransH-RS$^{\clubsuit }$	bern.	77	178	75.0	**53.6**	357	371	94.5	80.3
GTrans-DW$^{\clubsuit }$	unif.	147	256	63.4	44.1	*197*	*210*	92.2	78.4
GTrans-DW$^{\clubsuit }$	bern.	126	235	60.5	43.1	**166**	**180**	90.3	77.1
GTrans-SD$^{\clubsuit }$	unif.	66	207	75.1	50.6	234	247	92.9	79.1
GTrans-SD$^{\clubsuit }$	bern.	85	189	75.3	52.9	202	215	93.5	80.2
RosE$_{1l}$(ours)	unif.	79	233	**81.5**	50.4	271	284	**95.0**	**81.4**
RosE$_{2l}$(ours)	unif.	79	234	*80.4*	48.7	347	362	94.5	78.8
TransGH$^{\clubsuit }$	unif.	66	186	79.8	*54.0*	*179*	*191*	94.8	81.4
TransGH$^{\clubsuit }$	bern.	64	186	80.1	**54.1**	197	210	95.3	*81.6*
RotatE^16^	unif.	*40*	–	74.6	–	309	–	95.9	–
RatE^17^	unif.	$\mathbf{24}$	–	$\mathbf {89.8}$	–	180	–	$\mathbf {96.2}$	–
TimE^18^	unif.	45.9	–	87.9	–	259	–	*96.1*	–
TCIE^42^	unif.	48	–	*89.6*	–	260	–	95.8	–

The mark [$^{\clubsuit }$] denotes that the results are adapted from paper^22^. Other results are from the original papers.

In Table 5, the models can be attributed to two groups, the first one is based on linear transformations, and the second is based on non-linear transformations from the translation equation. For the models in the first group, the proposed model achieved the best result in the metric Hits@10 on datasets FB15k and was very close to the one on dataset WN18. However, these promising results achieved in TransGH were beneficial from the neural networks, and the model is no longer efficient and straightforward. If we ticked these models out, then the proposed model achieved SOTA over the pure linear transformation-based models such as TransE, TransH, TransR/CTransR, TransD, TransE-RS, and TransH-RS. When it turned to neural network models such as TransGH and GTrans, as well as non-linear transformation ones such as RotatE, RatE, and TimE, the proposed model lagged to different extents. Considering the proposed model only evaluated the $\ell _{1}$ norm in the scoring function, this result was more promising with low cost in time complexity compared to other translation-based models listed in the Table 1. From these experimental results, the models transformed from the translation equation by linear operation were incapable of overcoming the inherent limitations of the translation-based approach. Still, the weight and bias vectors indeed improved the expressive power of the model at a low cost, just as the Fig. 2c and Table 1 illustrated. Table 1 showed that the proposed model was in the same order of time complexity when the model was run in $\ell _2$ norm.

In Table 5, the models can be divided into two groups: those based on linear transformations and those based on non-linear transformations from the translation equation. For the first group, the proposed model achieved the best result in Hits@10 on the dataset FB15k and was very close to reaching the same result on the dataset WN18. However, these promising results were mainly due to the use of neural networks in TransGH, which made the model less efficient and straightforward. Excluding these models, the proposed model achieved SOTA performance over purely linear transformation-based models such as TransE, TransH, TransR/CTransR, TransD, TransE-RS, and TransH-RS. When compared to neural network models such as TransGH and GTrans or non-linear transformation models such as RotatE, RatE, and TimE, the proposed model lagged to varying degrees. However, considering that the proposed model only evaluated the $\ell _{1}$ norm in its scoring function and had low time complexity compared to other translation-based models listed in Table 1, this result is promising. These experimental results show that while linear transformations from translation equations are unable to overcome the inherent limitations of translation-based approaches, weight and bias vectors can improve expressive power at a low cost. This is illustrated in Fig. 2c and Table 1. Table 1 also shows that the proposed model has similar time complexity when run with an $\ell _2$ norm.

#### Performance on challenging datasets

The datasets FB15k237 and WN18RR are considered to be challenging and more closed to real-world KG. Translation-based models typically don’t report results on these datasets because they struggle to capture the sparse features present in them. Therefore, we must compare our results with those of factorization-based, logical rule-based, and neural network-based models.

The FB15k237 and WN18RR datasets are considered challenging and more representative of real-world knowledge graphs. Translation-based models typically do not report results on these datasets because they struggle to capture sparse features. As a result, the proposed models have to compare their results with those of factorization-based, logical rule-based, and neural network-based models.

**Table 6:** The result of challenging datasets.

Model	FB15K237				WN18RR			
	MRR	Hits@			MRR	Hits@		
	Filt.	1	3	10	Filt.	1	3	10
DistMust$^{\clubsuit }$	0.241	15.5	26.3	41.9	0.430	39.0	44.0	49.0
ComplEx$^{\clubsuit }$	0.247	15.8	27.5	42.8	0.440	41.0	46.0	51.0
ConvE$^{\clubsuit }$	0.325	23.0	35.6	50.1	0.430	40.0	44.0	52.0
TuckER$^{\spadesuit }$	**0.358**	**26.6**	**39.4**	**54.4**	0.470	*44.3*	48.2	52.6
PathRank$^{\spadesuit }$	0.087	7.4	9.2	11.2	0.189	17.1	20.0	22.5
NeuralLP$^{\spadesuit }$	0.237	17.3	25.9	36.1	0.381	36.8	38.6	40.8
DRUM$^{\spadesuit }$	0.238	17.4	26.1	36.4	0.382	36.9	38.8	41.0
MINERVA$^{\spadesuit }$	0.293	21.7	32.9	45.6	0.415	38.2	43.3	48.0
M-Walk$^{\spadesuit }$	0.232	16.5	24.3	–	0.437	41.4	–	44.5
RNNLogic$^{\spadesuit }$	0.288	20.8	31.5	44.5	0.455	41.4	47.5	53.1
RNNLogic1$^{\spadesuit }$	0.344	25.2	38.0	53.0	*0.483*	**44.6**	44.3	55.8
RotatE^16^	0.338	24.1	37.5	53.3	0.476	42.8	*49.2*	**57.1**
RatE^17^	0.344	*26.1*	*38.2*	*54.1*	**0.488**	44.1	**50.6**	**59.0**
TimE^18^	*0.346*	25.0	*38.2*	53.7	0.477	42.8	49.3	*57.7*
RotatPRH^43^	0.343	24.6	38.1	53.8	0.478	43.4	49.4	56.5
RosE$_{1l}$(ours)	0.282	19.2	31.4	48.6	0.203	2.7	35.1	48.6
RosE$_{2l}$(ours)	0.286	19.6	31.6	48.4	0.215	2.4	37.9	46.4

The mark [$^{\clubsuit }$] denotes the data from the paper^39^ and [$^{\spadesuit }$] from the paper^35^.

In Table 6, the models can be divided into two groups: the first group contains factorization-based, logical rule-based, and neural network-based models, while the second group includes models transformed from translation equations using non-linear operations. The experimental results show that the proposed model performed better than some of these models and was even competitive with specific neural network-based models such as NeuralLP^32^ and DRUM^33^. However, the proposed model still underperformed state-of-the-art (SOTA) results on challenging datasets, such as FB15k237 and WN18RR, when compared to other research lines and models that have been transformed using non-linear operations from translation equations. Models such as RotatE, RatE, and TimE achieved SOTA performance on challenging datasets within the translation-based research line. However, they operate in complex vector spaces and use non-linear transformations from translation equations, losing the simplicity and efficiency of TransE.

In this section, the proposed model was tested on three experiments on small, large, and challenging datasets to evaluate two versions of the proposed model, RosE, respectively. They illustrated different characters: on the first group of datasets, the proposed model achieved SOTA or competitive results; on the second group of datasets, the proposed model outperformed almost the existing translation-based models (especially the ones transformed from translation equation linearly), even competitive with some neural networks-based ones at a low cost; however, on the third group of datasets which were the challenging ones and almost no translation-based model transformed from translation equation by linear operation reported on them, the proposed model still outperformed most of them though not all. Furthermore, the models transformed from the translation equation by a non-linear operation still achieved outstanding performance not only on the large datasets FB15k and WN18 but also on the challenging ones FB15k237 and WN18RR. However, the proposed model still lagged behind those from other research lines, and even those transformed from the translation equation non-linearly in the translation-based research line on these datasets.

In this section, three experiments were conducted on small, large, and challenging datasets to evaluate two versions of the proposed model, RosE. These experiments demonstrated different characteristics: on the first group of datasets, the proposed model achieved SOTA or competitive results; on the second group of datasets, the proposed model outperformed most existing translation-based models (especially those transformed linearly from translation equations) and was even competitive with some neural network-based models at a low cost. However, on the third group of challenging datasets, where few linearly transformed translation-based models have reported results, the proposed model still outperformed most of them, but not all. Furthermore, models transformed from translation equations using non-linear operations achieved outstanding performance not only on large datasets such as FB15k and WN18 but also on challenging ones such as FB15k237 and WN18RR. However, the proposed model still lagged behind models from other research lines, as well as those transformed non-linearly from translation equations within the translation-based research line, on these datasets.

## Theoretical analyses

This section presents theoretical results concerning the capacity of the proposed model to capture reflexive, symmetric, and transitive relation features from knowledge graph data. These results are presented in the form of propositions and apply to both versions of the proposed model RosE. In the following proofs, $\mathbf{w}^1$, $\mathbf{w}^2$ and $\mathbf{w}^3$ represent weight vectors while $\mathbf{b}^1$, $\mathbf{b}^2$ and $\mathbf{b}^3$ represent bias vectors for the RosE$_{1l}$ model. For the RosE$_{2l}$ model, $\mathbf{w}^1_1$, $\mathbf{w}^2_1$, $\mathbf{w}^3_1$, $\mathbf{w}^1_2$, $\mathbf{w}^2_2$ and $\mathbf{w}^3_2$ represent weight vectors for entity pair and relation embeddings. These symbols are used only in this section to facilitate understanding of the proofs.

### Proposition 1

*In model RosE, if a relation* *r is reflexive and* $r\in \mathcal {R}$,* then the fact* $(e, r, e)\in \Delta$* and* $\Delta \subset \mathcal {G}$* hold*.

### Proof

If a relation is reflexive, then it implies that the fact $(e,r,e)\in \Delta$ is included in the KG. There are two cases for the model RosE.

**RosE**$_{1l}$ According to the Eq. (8), there exists an equation $\mathbf{w}\circ (\mathbf{e}-\mathbf{e})=-\mathbf{b}$ since the relation is reflexive. It is easy to find that if $\mathbf{b}=\mathbf{0}^d$ holds, then $\mathbf{w}$ can be an arbitrary vector which can be learned from the data towards a suitable one. Therefore, $(e,r,e)\in \Delta$ and $\Delta \subset \mathcal {G}$ hold in the model RosE$_{1l}$.

**RosE**$_{2l}$ In this case, According to the Eq. (9), the equations $\mathbf{w}_{1}\circ (\mathbf{e}-\mathbf{e})=-\mathbf{w}_{2}\mathbf{r}$ must hold. Therefore, $\mathbf{w}_{2}\circ \mathbf{r}=\mathbf{0}^d$ and $\mathbf{w}_{2}\circ \mathbf{r}=\mathbf{0}^d$ also hold. Because the relation embedding $\mathbf{r}$ is not equal to $\mathbf{0}^d$ but $\mathbf{w}_{2}\circ \mathbf{r} = \mathbf{0}^d$ and this condition is easy to be satisfied since it is an arbitrary vector and these solutions can be learned from data. Therefore, $(e,r,e)\in \Delta$ and $\Delta \subset \mathcal {G}$ hold in the model RosE$_{2l}$.

In sum, the model RosE is capable of capturing the features of reflexive relation due to $\Delta \subset \mathcal {G}$. *□*

### Proposition 2

*In model RosE, if a relation* *r is symmetric, then a fact* $(h,r,t)\in \Delta \Leftrightarrow (t,r,h)\in \Delta$* and* $\Delta \subset \mathcal {G}$.* If a relation* *r is reflexive, then it is also symmetric*.

### Proof

If a relation is symmetric, then it implies that the facts (*h*, *r*, *t*) and (*t*, *r*, *h*) are included in KG. There are two cases in the model RosE.

**RosE**$_{1l}$ In the model RosE$_{1l}$, according to the Eq. (8), the equations $\mathbf{w}^{1}\circ (\mathbf{t}-\mathbf{h})=-\mathbf{b}^{1}$ and $\mathbf{w}^{2}\circ (\mathbf{h}-\mathbf{t})=-\mathbf{b}^{2}$ must hold since the relation *r* is symmetric. If the relation is not reflexive, then the symmetric relation features can also be expressed in this model. In this case, $\mathbf{h} \ne \mathbf{t}$ and we can get the equation $\mathbf{w}^{1}\circ \mathbf{b}^{2}+\mathbf{w}^{2}\circ \mathbf{b}^{1}=\mathbf{0}^d$. Since $\mathbf{w}^{1}$, $\mathbf{b}^{2}$, $\mathbf{w}^{2}$ and $\mathbf{b}^{2}$ are arbitrary vectors, this equation is always can be satisfied easily. On the contrary, if relation *r* is reflexive, then $\mathbf{h} = \mathbf{t}$ holds in this sense. As a result, $\mathbf{b}^1 = \mathbf{0}^d$ and $\mathbf{b}^2 = \mathbf{0}^d$ is the reasonable solutions for this case and $\mathbf{w}^1$ and $\mathbf{w}^2$ are also arbitrary vectors. Therefore, a fact $(h,r,t)\in \Delta \Leftrightarrow (t,r,h)\in \Delta$ hold and $\Delta \subset \mathcal {G}$.

Furthermore, if $\mathbf{b}^{1} = \mathbf{0}^d$ and $\mathbf{b}^{2} = \mathbf{0}^d$, then this is also a solution for the case where relation *r* is symmetric. Similarly, $\mathbf{w}^{1}$ and $\mathbf{w}^{2}$ can be any arbitrary vectors. This means that in the RosE$_{1l}$ model, if a relation is reflexive, then it must also be symmetric. This reveals that the RosE$_{1l}$ model has a restriction: if a relation is reflexive, then it must also be symmetric.

**RosE**$_{2l}$ Similarly, for the model RosE$_{2l}$, according to the Eq. (9), the equations $\mathbf{w}^{1}_{1}\circ (\mathbf{t}-\mathbf{h})=-\mathbf{w}^{1}_{2}\mathbf{r}$ and $\mathbf{w}_{2}^{2}\circ (\mathbf{h}-\mathbf{t})=-\mathbf{w}^{2}_{2}\mathbf{r}$ must hold. If the relation *r* is not reflexive, then it means $\mathbf{h}\ne \mathbf{t}$ holds. Therefore, the equation $\mathbf{w}_{1}^{1}\circ \mathbf{w}_{2}^{2}+\mathbf{w}_{2}^{1}\circ \mathbf{w}_{1}^{2}=\mathbf{0}$ holds in this case. These conditions are easy to satisfy. In sum, a fact $(h,r,t)\in \Delta \Leftrightarrow (t,r,h)\in \Delta$ and $\Delta \subset \mathcal {G}$ must hold. If the relation is reflexive, then $\mathbf{h} = \mathbf{t}$ must hold. Furthermore, because relation embedding $\mathbf{r}\ne \mathbf{0}$ always holds, $\mathbf{w}^{1}_{2} = \mathbf{0}^d$ and $\mathbf{w}^{2}_{2} = \mathbf{0}^d$ are true in this case. As a result, $\mathbf{w}^{2}_{1}$ and $\mathbf{w}^{2}_{2}$ are arbitrary real vectors and thus the equation is easy to satisfy.

Additionally, if relation *r* is reflexive and $\mathbf{w}^{1}{2} = \mathbf{0}^d$, then $\mathbf{w}^{2}{2} = \mathbf{0}^d$ is also a solution to the equation. In this case, $\mathbf{w}^{1}{1}$ and $\mathbf{w}^{1}{2}$ can be any arbitrary vectors and are also solutions to the equation. In other words, if a relation is reflexive, then it must also be symmetric.*□*

### Corollary 1

*RosE is capable of expressing the symmetric relation with the limitation that if a relation is reflexive, then it is also symmetric*.

### Proposition 3

*In the model RosE, if a relation* *r is transitive, then the reasoning* $(i,r,j)\wedge (j,r,k)\Rightarrow (i,r,k)$* holds i.e.*, $(i,r,k)\in \Delta$* and* $\Delta \subset \mathcal {G}$.* If a relation* *r is reflexive, then it is also transitive*.

### Proof

If a relation is transitive, it implies that if the facts (*i*, *r*, *j*) and (*j*, *r*, *k*) hold simultaneously in a knowledge graph (KG), then (*i*, *r*, *k*) must also hold where *i*, *j* and *k* are entities and $\mathbf{i}$, $\mathbf{j}$ and $\mathbf{k}$ are their corresponding embeddings. For the two versions of the RosE model, there are two cases to consider.

**RosE**$_{1l}$ In the model RosE$_{1l}$, according to the Eq. (8), if the relation *r* is transitive, then the equations $\mathbf{w}^{1}\circ (\mathbf{j}-\mathbf{i})=-\mathbf{b}^{1}$ and $\mathbf{w}^{2}\circ (\mathbf{k}-\mathbf{j})=-\mathbf{b}^{2}$, $\mathbf{w}^{3}\circ (\mathbf{k}-\mathbf{i})=-\mathbf{b}^{3}$ must also hold since relation *r* is transitive. If relation *r* is not reflexive, then the equations $\mathbf{i} \ne \mathbf{j} \ne \mathbf{k}$ and $\mathbf{j}-\mathbf{i} = -\frac{\mathbf{b}^{1}}{\mathbf{w}^{1}}$ and $\mathbf{k}-\mathbf{j} = -\frac{\mathbf{b}^{2}}{\mathbf{w}^{2}}$ hold. Therefore, the equation $\mathbf{k}-\mathbf{i} = -(\frac{\mathbf{b}^{1}}{\mathbf{w}^{1}}+\frac{\mathbf{b}^{2}}{\mathbf{w}^{2}})=-\frac{\mathbf{w}^{1}\circ \mathbf{b}^{2}+\mathbf{w}^{2}\circ \mathbf{b}^{1}}{\mathbf{w}^{1}\circ \mathbf{w}^{2}}$ holds. If the fact $(i,r,k)\in \Delta$ holds and the equation $\mathbf{k}-\mathbf{i} = -\frac{\mathbf{b}^{3}}{\mathbf{w}^{3}}$ is also satisfied, additionally, if and only if the weight and bias vectors satisfy the equation $\mathbf{b}^{1}\circ \mathbf{w}^{2}\circ \mathbf{w}^{3} + \mathbf{w}^{1}\circ \mathbf{b}^{2}\circ \mathbf{w}^{3} + \mathbf{w}^{1}\circ \mathbf{w}^{2}\circ \mathbf{b}^{3} = \mathbf{0}^d$, then the proposition holds. The possible solutions for this equation are infinite. Therefore, if a relation *r* is transitive, then the reasoning $(i,r,j)\wedge (j,r,k)\Rightarrow (i,r,k)$ holds i.e. $(i,r,k)\in \Delta$ and $\Delta \subset \mathcal {G}$.

If relation *r* is reflexive, then the equation $\mathbf{i} = \mathbf{j} = \mathbf{k}$ holds and all three can be represented by $\mathbf{e}$. The solutions to this equation satisfy the conditions $\mathbf{b}^{1} = \mathbf{0}^d$, $\mathbf{b}^{2} = \mathbf{0}^d$ and $\mathbf{b}^{3} = \mathbf{0}^d$. As for $\mathbf{w}^{1}$, $\mathbf{w}^{2}$ and $\mathbf{w}^{3}$, they can be any arbitrary vectors. In other words, if a relation *r* is reflexive, then it must also be transitive.

**RosE**$_{2l}$ In the model RosE$_{2l}$, according to the Eq. (9), if the relation *r* is transitive, then the equations $\mathbf{w}_{1}^{1}\circ (\mathbf{j}-\mathbf{i})=-\mathbf{w}^{1}_{2}\circ \mathbf{r}$, $\mathbf{w}_{1}^{2}\circ (\mathbf{k}-\mathbf{j})=-\mathbf{w}_{2}^{2}\circ \mathbf{r}$ and $\mathbf{w}_{1}^{3}\circ (\mathbf{k}-\mathbf{j})=-\mathbf{w}_{2}^{3}\circ \mathbf{r}$ must hold. If the relation *r* is not reflexive, then the equations $\mathbf{j}-\mathbf{i} = -\frac{\mathbf{w}^{1}_{2}\circ \mathbf{r}}{\mathbf{w}_{1}^{1}}$ and $\mathbf{k}-\mathbf{j} = -\frac{\mathbf{w}^{2}_{2}\circ \mathbf{r}}{\mathbf{w}_{1}^{2}}$ also hold. Therefore, the equation $\mathbf{k}-\mathbf{i} = -(\frac{\mathbf{w}^{1}_{2}\circ \mathbf{r}}{\mathbf{w}_{1}^{1}} + \frac{\mathbf{w}^{2}_{2}\circ \mathbf{r}}{\mathbf{w}_{1}^{2}})$ holds. The condition can be expressed as $\mathbf{w}_{1}^{1}\circ \mathbf{w}_{1}^{2}\circ \mathbf{w}_{2}^{3}+\mathbf{w}_{2}^{1}\circ \mathbf{w}_{1}^{2}\circ \mathbf{w}_{2}^{3}+\mathbf{w}_{2}^{1}\circ \mathbf{w}_{2}^{2}\circ \mathbf{w}_{1}^{3} = \mathbf{0}^d$ and easy to be satisfied. This equation has infinite solutions. Formally, if a relation *r* is transitive, then the reasoning $(i,r,j)\wedge (j,r,k)\Rightarrow (i,r,k)$ holds i.e. $(i,r,k)\in \Delta$ and $\Delta \subset \mathcal {G}$.

Given the relation *r* is reflexive, the entity embeddings related equation $\mathbf{i} = \mathbf{j} = \mathbf{k} =\mathbf{e}$ holds, we get the equations $\mathbf{w}_{1}^{1}\circ (\mathbf{e}-\mathbf{e})=-\mathbf{w}^{1}_{2}\circ \mathbf{r}$, $\mathbf{w}_{1}^{2}\circ (\mathbf{e}-\mathbf{e})=-\mathbf{w}_{2}^{2}\circ \mathbf{r}$, $\mathbf{w}_{1}^{3}\circ (\mathbf{e}-\mathbf{e})=-\mathbf{w}_{2}^{3}\circ \mathbf{r}$, $\mathbf{w}_{1}^{1} = \mathbf{0}^d$, $\mathbf{w}_{1}^{2} = \mathbf{0}^d$ and $\mathbf{w}_{1}^{3} = \mathbf{0}^d$ since $\mathbf{r} \ne \mathbf{0}^d$ holds. What’s more, $\mathbf{w}_{2}^{1}$, $\mathbf{w}_{2}^{2}$ and $\mathbf{w}_{2}^{3}$ are arbitrary vectors. These solutions are also suitable for the equation $\mathbf{w}_{1}^{1}\circ \mathbf{w}_{1}^{2}\circ \mathbf{w}_{2}^{3}+\mathbf{w}_{2}^{1}\circ \mathbf{w}_{1}^{2}\circ \mathbf{w}_{2}^{3}+\mathbf{w}_{2}^{1}\circ \mathbf{w}_{2}^{2}\circ \mathbf{w}_{1}^{3} = \mathbf{0}^d$. As a result, we can conclude that if a relation *r* is reflexive, then it is also transitive. *□*

### Corollary 2

*The RosE model is capable of expressing transitive relations with the limitation that if a relation is reflexive, then it must also be transitive*.

### Proposition 4

*In the RosE model, if an entity* *h has relation* *r with every entity in* *Δ** and another entity* *h'** has relation* *r with at least one entity in* $\Delta \subset \mathcal {E}$,* then* *h'** also has relation* *r with every entity in* *Δ*.

### Proof

Let *h* be an arbitrary head entity that has a relation *r* between two arbitrary entities $t_1$, $t_2\in \Delta$. Similarly, let *h'* be an arbitrary head entity that has relation *r* between the entities $t_1$, $t_2\in \Delta$.

**RosE**$_{1l}$ In model RosE$_{1l}$, according to the Eq. (8), there exist the equations $\mathbf{w}^1\circ (\mathbf{t}_1 - \mathbf{h}) = -\mathbf{b}^1$ and $\mathbf{w}^2\circ (\mathbf{t}_2 - \mathbf{h}) = -\mathbf{b}^2$. If equations $\mathbf{w}^1 = \mathbf{0}^d$, $\mathbf{w}^2 = \mathbf{0}^d$ and $\mathbf {w'}^1 = \mathbf{0}^d$ hold, then solutions $\mathbf{b}^1 = \mathbf{0}^d$, $\mathbf{b}^2 = \mathbf{0}^d$ and $\mathbf {b'}^1 = \mathbf{0}^d$ also can make the equations hold. In this case, $\mathbf {w'}^2 = \mathbf{0}^d$ and $\mathbf {b'}^2 = \mathbf{0}^d$ are also true i.e. equation $\mathbf {w'}^2\circ (\mathbf{t}_2 - \mathbf {h'}) = \mathbf {b'}^2$ also holds and $(h',r,t_2)\in \Delta$. If equations $\mathbf{w}^1 \ne \mathbf{0}^d$, $\mathbf{w}^2 \ne \mathbf{0}^d$ and $\mathbf {w'}^1 \ne \mathbf{0}^d$ hold, then equation $\mathbf{t}_1 = \mathbf{t}_2 + \frac{\mathbf{b}^2}{\mathbf{w}^2} - \frac{\mathbf{b}^1}{\mathbf{w}^1}$ holds. For the fact $(h',r,t_1)\in \Delta$, there exists equation $\mathbf {w'}^1\circ (\mathbf{t}_1 - \mathbf {h'}) = \mathbf {b'}^1$, then equation $\mathbf {w'}^1\circ (\mathbf{t}_2 + \frac{\mathbf{b}^2}{\mathbf{w}^2} - \frac{\mathbf{b}^1}{\mathbf{w}^1} - \mathbf {h'}) = -\mathbf {b'}^1$ holds. Further, it can be transformed as $\mathbf {w'}^1\circ (\mathbf{t}_2 - \mathbf {h'}) = \mathbf {w'}^1\circ (\frac{\mathbf{b}^1}{\mathbf{w}^1} - \frac{\mathbf{b}^2}{\mathbf{w}^2})$. Let equations $\mathbf {w'}^2 = \mathbf {w'}^1$ and $\mathbf {b'}^2 = \mathbf {w'}^1\circ (\frac{\mathbf{b}^1}{\mathbf{w}^1} - \frac{\mathbf{b}^2}{\mathbf{w}^2})$ hold, and the equation $\mathbf {w'}^2\circ (\mathbf{t}_2 - \mathbf {h'}) = -\mathbf {b'}^2$ also holds. Therefore, the fact $(h',r,t_2)\in \Delta$ holds.

**RosE**$_{2l}$ In model RosE$_{2l}$, according to the Eq. (9), there exist equations $\mathbf{w}^1_1(\mathbf{t}_1 - \mathbf{h}) = -\mathbf{w}_2^1\mathbf{r}$ and $\mathbf{w}^2_1(\mathbf{t}_2 - \mathbf{h}) = -\mathbf{w}_2^2\mathbf{r}$. If another entity *h'* has relation *r* with $t_1$, then there exists an equation $\mathbf {w'}^1_1(\mathbf{t}_1 - \mathbf {h'}) = -\mathbf {w'}_2^1\mathbf{r}$. Similarly, if the equations $\mathbf{w}_1^1 = \mathbf{0}^d$, $\mathbf{w}_1^2 = \mathbf{0}^d$ and $\mathbf {w'}_1^1 = \mathbf{0}^d$ hold, then equations $\mathbf{w}_2^1 = \mathbf{0}^d$, $\mathbf{w}_2^2 = \mathbf{0}^d$ and $\mathbf {w'}_2^1 = \mathbf{0}^d$ also can satisfy the condition. If equations $\mathbf{w}_1^1 \ne \mathbf{0}^d$, $\mathbf{w}_1^2 \ne \mathbf{0}^d$ and $\mathbf {w'}_1^1 \ne \mathbf{0}^d$ hold, then equation $\mathbf{t}_1 = \mathbf{t}_2 + \frac{\mathbf{w}_2^2}{\mathbf{w}_2^2} - \frac{\mathbf{w}_2^1}{\mathbf{w}_1^1}$ holds. For the fact $(h',r,t_1)\in \Delta$, there exists relationship $\mathbf {w'}_1^1\circ (\mathbf{t}_1 - \mathbf {h'}) = \mathbf {w'}_2^1$, therefore, the equivalent equation is $\mathbf {w'}_1^1\circ (\mathbf{t}_2 + \frac{\mathbf{w}_2^2}{\mathbf{w}_1^2} - \frac{\mathbf{w}_2^1}{\mathbf{w}_1^1} - \mathbf {h'}) = -\mathbf {w'}_2^1$. Further, it can be transformed as $\mathbf {w'}_1^1\circ (\mathbf{t}_2 - \mathbf {h'}) = \mathbf {w'}_1^1\circ (\frac{\mathbf{w}_2^1}{\mathbf{w}_1^1} - \frac{\mathbf{w}_2^2}{\mathbf{w}_1^2})$. Given equations $\mathbf {w'}_1^2 = \mathbf {w'}_1^1$ and $\mathbf {w'}_2^2 = \mathbf {w'}_1^1\circ (\frac{\mathbf{w}_2^1}{\mathbf{w}_1^1} - \frac{\mathbf{w}_1^2}{\mathbf{w}_1^2})$ hold, the equation $\mathbf {w'}_1^2\circ (\mathbf{t}_2 - \mathbf {h'}) = -\mathbf {w'}_2^2$ also holds. Therefore, the fact $(h',r,t_2)\in \Delta$ holds.*□*

From the above proofs, it can be safely concluded that the RosE model is capable of expressing reflexive, symmetric, and transitive relations. This makes it more expressive than the baseline model TransE. However, like other translation-based models pointed out by Kazemi et al.^15^, RosE has the restriction that if a relation *r* is reflexive, then it must also be symmetric and transitive. Furthermore, the proposed model has the limitation that if an entity $e_1$ has relation *r* with every entity *e'* in $\mathcal {E}$ and another entity $e_2$ has relation *r* with at least one entity $e'\in \mathcal {E}$, then $e_2$ must also have relation *r* with every entity in $\mathcal {E}$. Although the proposed model is more general than those in^15^, it still has all three limitations. These theoretical results may seem disappointing. However, according to the proof of **Proposition 2** (refer to A), the term *variants of translating approaches* refers only to those transformed linearly from translation equations and not non-linearly. In other words, not all variants of translation approaches are included. This point is clarified in this work.

### Corollary 3

*Like linearly transformed translation-based models such as TransE, TransH, and TransR, the RosE model is also subject to the three limitations mentioned above. However, these limitations do not apply to models transformed non-linearly from translation equations*.

In this sense, linear transformations are usually unable to overcome the three limitations inherent in translation equations. These limitations are intrinsic to translation equations and cannot be overcome by linear transformations or the introduction of additional degrees of freedom. As a result, numerous models have been proposed that utilize non-linear transformations or other techniques to circumvent these limitations.

## Discussion

It is known that the proposed model achieves SOTA performance in the research line of translation-based models at a lower cost than previous ones on most datasets in Table 2. The proposed model pushes the boundary of KGC to a new level in this research line, as it applies linear operations to most datasets, transforming models from translation equations. It is competitive with other approaches on widely used datasets. However, this conclusion does not hold for other research lines, such as factorization-based, logical rules-based, and neural networks-based approaches on challenging datasets. In sum, the models transformed from translation equation linearly (including the proposed model RosE in this work) share the shortcomings such as reflexive, symmetric, and transitive relations in the KGC task. This may be due to the model’s weakness in handling long-tail effects.

Given that there are few translation-based models transformed from the translation equation by linear operations that report results on datasets FB15k237 and WN18RR, we can reasonably speculate that such approaches may struggle to cope with long-tail effects, despite their outstanding performance on other datasets. This may guide us in choosing reasonable models for real-world applications.

KG is a dataset with graph structure; therefore, they can be stored in the form of an attribute graph. The approach LCAAG^44^ can potentially benefit from effective KGC within a group entity after clustering. In this way, the proposed model can be more interpretable^45^ in the process of KGC.

Furthermore, it is difficult to determine the effects of removing relation representation from RosE$_{1l}$ on the proposed model. Numerous studies have demonstrated that models transformed by linear transformations of translation equations cannot overcome the inherent limitations of translation equations.

### Limitations of the study

Although the proposed model improves performance on widely used datasets and achieves SOTA or competitive results at a lower cost, it does not consistently achieve SOTA performance on challenging datasets compared to other research lines. The model has demonstrated a promising capability in capturing features from datasets, including reflexive, symmetric, and transitive relations in knowledge graphs.

However, there are several areas for future work: (1) Are there any restrictions on models from other research lines, such as factorization-based, logical rules-based, and neural networks-based? (2) Is it possible to deal with the long-tail effect of datasets in translation-based models transformed from translation equations by linear operations? (3) If linear models of KGC become fully lagging, how can we design a simpler and more efficient model? (4) If a model captures key features from datasets, it will win in the trade-off between expressive power and expensiveness. The question is how to determine which are the key features of a KG.

## Methods

Two linear variants of the model RosE are proposed. In this section, the details of how RosE was constructed are presented. $\mathbf{w}$, $\mathbf{w}_{1}$, and $\mathbf{w}_{2}$ are used to denote the weight vectors of entity pairs, and $\mathbf{b}$ is used to denote the bias vector.

### Representation

A classic translation embedding model serves as the inspiration for the proposed model. It projects each symbolic element in KG, such as entity and relation, into a low-dimensional vector space and performs KGC in this space. Following the principle of additive compositionality, it ranks the candidate entities based on the scores computed by a scoring function. To obtain these scores, it randomly initializes *d*-dimensional vectors for each symbolic entity and relation in KG and denotes them as $\mathbf{h}$, $\mathbf{t}$, and $\mathbf{r}$, similar to the model TransE.

**Fig. 3:**
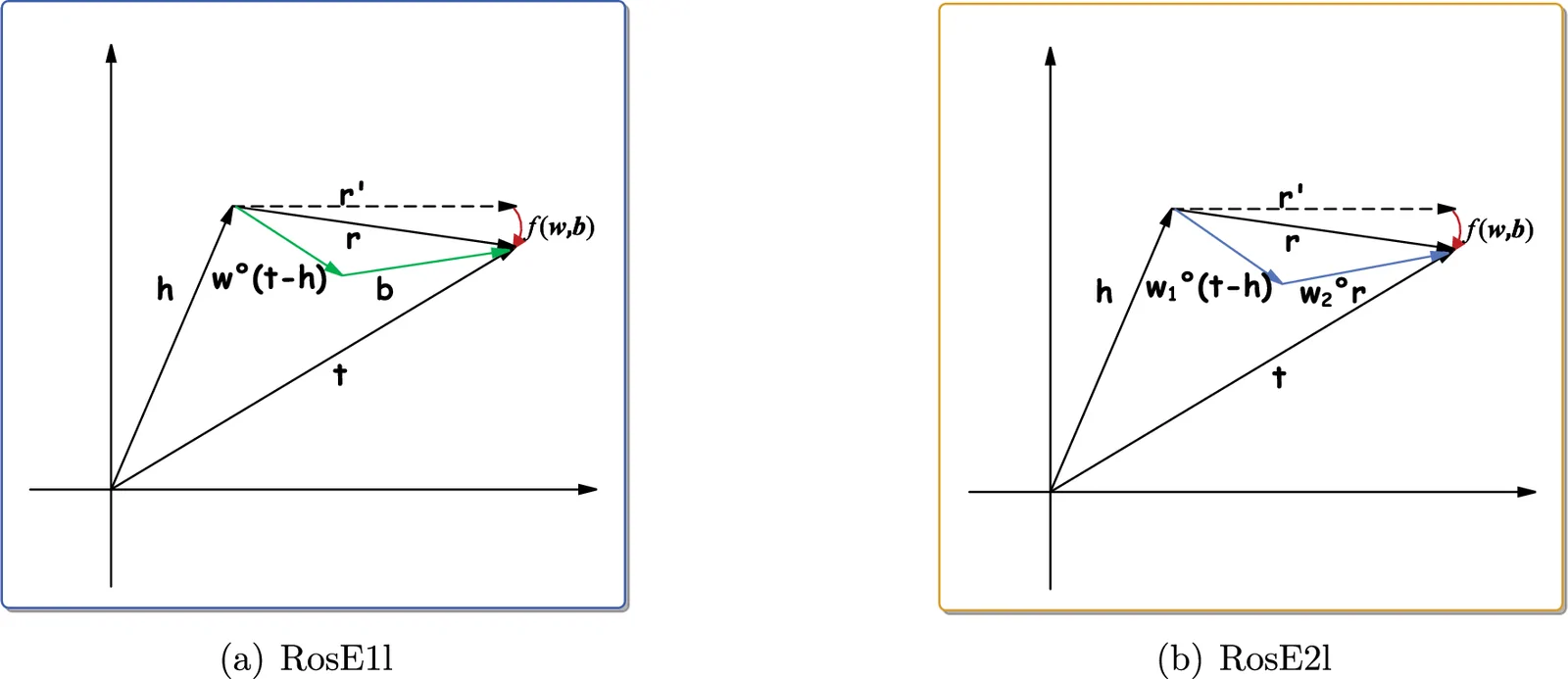
These Fig. 2 illustrates the geometric meaning of RosE$_{1l}$ and RosE$_{2l}$. It shows how the two variants of RosE reduce the gap $\mathbf{g}$ by rotating and scaling the entity and relation embeddings to align the actual relation embedding $\mathbf{r}$ with the expected one $\mathbf{r}'$.

#### RosE$_{1l}$

To explain how to rotate and scale the translation equation to match a specific relation, and how to apply a linear transformation to the translation equation, the derivation of the scoring criterion for model RosE$_{1l}$ is shown in Appendix B. Thus, two degrees of freedom are introduced into the translation equation to enhance its expressive power. The modified translation equation is

8 $$\begin{aligned} \mathbf{w}\circ (\mathbf{t} - \mathbf{h}) = - \mathbf{b}. \end{aligned}$$

As can be seen from Eq. (8), it is a linear equation. In summary, the scoring criterion $\vert \mathbf{w}\circ (\mathbf{t} - \mathbf{h}) + \mathbf{b} \vert ^{l}$ for model RosE$_{1l}$ is obtained from the translation equation by applying a linear transformation, which is named *main component*. The derivation shows that it is a single linear function.

RosE$_{1l}$ is the first variant of the proposed model RosE. In the derivation process, it is assumed that $\mathbf{r}\ne \mathbf{0}^d$ according to Appendix B. This means that none of the elements in vector $\mathbf{r}$ is zero since the division is element-wise. After a series of linear operations, the embedding $\mathbf{r}$ vanishes from the final equation. From a training perspective, the condition of $\mathbf{r}\ne \mathbf{0}^d$ does not affect the model performance so that it can be ignored. Thus, we only need to focus on the function $\mathbf{y} = (\mathbf{t} - \mathbf{h})\circ \mathbf{w}+\mathbf{b}$, which is a simple linear function. It successfully reduces the gap between entity pair and relation embeddings as shown in Fig. 3a and b . However, this model lacks relation embedding $\mathbf{r}$ in its scoring criterion in Appendix B. As a result, it loses the ability to capture and learn the features of relations in KG, which is a major drawback. Therefore, we need a *correction term* to address this issue, which is discussed in detail in section “Correction Term”.

#### RosE$_{2l}$

Similarly, to align the translation equation with a specific relation linearly and increase the expressive power of the scoring criterion, we derive the main component of model RosE$_{2l}$ in Appendix C. We also introduce two degrees of freedom into the modified translation equation in the form of weights as follows,

9 $$\begin{aligned} \mathbf{w}_{1}\circ (\mathbf{h} - \mathbf{t}) = -\mathbf{w}_{2}\circ \mathbf{r}. \end{aligned}$$

Thus, the main component of model RosE$_{2l}$ $\vert \mathbf{w}_{1}\circ (\mathbf{t} - \mathbf{h}) + \mathbf{w}_{2}\circ \mathbf{r}\vert ^{l}$ is the sum of two linear functions obtained from the translation equation by applying linear transformations as shown in Appendix C.

These transformations reveal that the main components of both variants of RosE are linear functions. Therefore, they also preserve the linearity property as illustrated in Fig. 3a and b . Every step is an equivalent transformation. This means that their effects on the transformations cancel out. Hence, these vector variables do not influence the outcomes of the models, and they are only auxiliary variables used for derivation.

### Correction term

From the derivation of the main component of model RosE$_{1l}$ in Appendix B, we observe that the relation embedding $\mathbf{r}$ vanishes after the transformation. This implies that the model loses its ability to express the relations in KG. To retain the relation features in the learning process, we add a *correction term* $\delta _{1} \vert \mathbf{h} + \mathbf{r} -\mathbf{t}\vert ^{l}$ to form the scoring function of this model. For a fair comparison with the other variant of RosE, we add a similar term $\delta _{2} \vert \mathbf{h} + \mathbf{r} -\mathbf{t}\vert ^{l}$ with a different coefficient.

### Scoring function

One of the simplest translation-based models is TransE, which uses an additive compositionality assumption^20^ for its scoring function, as shown in Eq. (2). However, this simplicity also limits its expressive power. To overcome this limitation, two variants of TransE are presented that incorporate two forms of transformation. Unlike TransE, the proposed models have two components. The scoring function of RosE$_{1l}$ is defined as follows,

10 $$\begin{aligned} f_{1l}(h,r,t) = \vert \mathbf{w}\circ (\mathbf{t} - \mathbf{h}) + \mathbf{b} \vert ^{l} + \delta _{1} \vert \mathbf{h} + \mathbf{r} -\mathbf{t}\vert ^{l}. \end{aligned}$$

The subscript 1*l* represents the model RosE$_{1l}$, which uses a single linear function as its main component in the scoring function and $l=\ell {1}$ for simplicity. The scoring function consists of two components. The first component $\vert \mathbf{w}\circ (\mathbf{t} - \mathbf{h}) + \mathbf{b} \vert ^{l}$, termed the *main component*, is a linear transformation of the translation equation as shown in Fig. 3a. The second component $\delta _{1} \vert \mathbf{h} + \mathbf{r} -\mathbf{t}\vert ^{l}$ is called the *correction term*, which is explained in section “Correction Term”. Additionally, there is another version of the scoring function that consists of two linear functions as follows,

11 $$\begin{aligned} f_{2l}(h,r,t) = \vert \mathbf{w}_{1}\circ (\mathbf{t} - \mathbf{h}) + \mathbf{w}_{2}\circ \mathbf{r}\vert ^{l} + \delta _{2} \vert \mathbf{h} + \mathbf{r} -\mathbf{t}\vert ^{l}. \end{aligned}$$

The subscript 2*l* represents RosE$_{2l}$, which is a linear transformation of the translation equation. The models RosE1*l* and RosE$_{2l}$ belong to the family of *RosE*. As shown in Fig. 3a, TransE is unable to express the features underlying the KG fully. To address this limitation, two versions of the RosE model are proposed, illustrated in Fig. 3a and b . The green dotted line represents the real relation vector. In RosE$_{1l}$, this result is achieved by rotating and scaling the embedding ($\mathbf{t} - \mathbf{h}$) and adding a learnable bias vector $\mathbf{b}$. In RosE$_{2l}$, the same goal is achieved by rotating and scaling both the embedding ($\mathbf{t} - \mathbf{h}$) and $\mathbf{r}$. Thus, both versions of the model achieve their goal in similar ways. For a better understanding of these models, the detailed derivations of their scoring functions are provided in the appendix.

### Loss and optimization

The loss functions for the two versions of RosE correspond to their respective scoring functions and follow the TransE paradigm. As a result, they can be constructed in the following manner,

12 $$\begin{aligned} \mathcal {L}_{1l} = \sum _{(h,r,t)\in \Delta }\sum _{(h',r,t')\in \Delta '}\max \{0,\gamma + f_{1l}(h,r,t) + f_{1l}^{'}(h',r,t')\}. \end{aligned}$$

This loss function is for the model RosE$_{1l}$, and the following one is for the model RosE$_{2l}$. That is,

13 $$\begin{aligned} \mathcal {L}_{2l} = \sum _{(h,r,t)\in \Delta }\sum _{(h',r,t')\in \Delta '}\max \{0,\gamma + f_{2l}(h,r,t) + f_{2l}(h',r,t')\}, \end{aligned}$$

where the function $\max \{0,x\}$ is a function for ensuring the results are no less than 0.

### Regularization terms and objective functions

The inclusion of weights and biases in the translation equation enhances the model’s expressiveness. To prevent overfitting, each model has two regularization terms. $h_{1l}(\mathbf{w},\mathbf{b})$ is the regularization term for the RosE$_{1l}$ model,

14 $$\begin{aligned} \text {h}_{1l}(\mathbf{w},\mathbf{b}) = \mu _{1}\vert \mathbf{w}\vert ^{l} + \theta _{1}\vert \mathbf{b}\vert ^{l}. \end{aligned}$$

Similarly, $h_{2l}(\mathbf{w}_1,\mathbf{w}_2)$ is the regularization term for the model RosE$_{2l}$,

15 $$\begin{aligned} \text {h}_{2l}(\mathbf{w}_1,\mathbf{w}_2) = \mu _{2}\vert \mathbf{w}_{1}\vert ^{l} + \theta _{2}\vert \mathbf{w}_{2}\vert ^{l}, \end{aligned}$$

where $\mu _{1},\theta _{1},\mu _{2}$ and $\theta _{2}$ are hyperparameters to which the models are sensitive and *l* represents the $\ell _{2}$ norm. The objective function is composed of the loss function and the regularization term. The corresponding objective functions for the RosE$_{1l}$ and RosE$_{2l}$ models are denoted as $\mathcal {O}_{1l}$ and $\mathcal {O}_{2l}$ respectively,

16 $$\begin{aligned} \mathcal {O}_{1l} = \mathcal {L}_{1l} + \text {h}_{1l}(\mathbf{w},\mathbf{b}) \end{aligned}$$

and

17 $$\begin{aligned} \mathcal {O}_{2l} = \mathcal {L}_{2l} + \text {h}_{2l}(\mathbf{w}_1,\mathbf{w}_2). \end{aligned}$$

The final step is to minimize the two objective functions for the two versions of the RosE model. These objective functions are non-convex and are optimized using SGD.

## Data Availability

The data and codes reported in this paper is publicly available on GitHub https://github.com/gzupanda/RosE

## Appendix A: Proposition 2

This proposition is highly relevant to this study and is taken from the paper^15^ for better understanding.

**Proposition 2 **FTransE is not fully expressive and has the following restrictions. R1: If a relation r is reflexive on $\Delta \in \mathcal {E}$, r must also be symmetric on *Δ*, R2: If a relation r is reflexive on $\Delta \in \mathcal {E}$, r must also be transitive on *Δ*, and R3: If entity $e_1$ has relation r with every entity in $\Delta \in \mathcal {E}$, and then $e_2$ must have the relation r with every entity in *Δ*.

## Appendix B: Main component of RosE$_{1l}$

This derivation focuses on certain elementary transformations based on the translation equation. In this process, let $\mathbf {b'}$ and $\mathbf{y}$ denote *d*-dimensional vectors and let *κ* be a real number used as a coefficient to adjust the scale of a vector.

$$\begin{aligned}&\text {According to the additive compositionality principle, it goes}\\&\because \mathbf{h} + \mathbf{r} \approx \mathbf{t},\\&\therefore \mathbf{t} - \mathbf{h} \approx \mathbf{r}.\\&\text {Let vector }\mathbf{b}^{'} \text { be a real vector and } \kappa \text { be an arbitrary scalar. }\\&\therefore \mathbf{r} = \kappa (\mathbf{t} - \mathbf{h}) + \mathbf{b}^{'}.\\&\text {If the condition }\mathbf{r}\ne \mathbf{0}^d \text { holds, then it can be divided by }\mathbf{r}\\&\text { element}\text {-}\text {wisely in two sides.}\\&\because \mathbf{1}^d =\frac{\kappa (\mathbf{t} - \mathbf{h})}{\mathbf{r}} + \frac{\mathbf{b}^{'}}{\mathbf{r}},\\&\therefore \mathbf{0}^d =\frac{\kappa }{\mathbf{r}}\circ (\mathbf{t} - \mathbf{h})+ (\frac{\mathbf{b}^{'}}{\mathbf{r}} - \mathbf{1}^d). \\&\text {If }\mathbf{y}=\mathbf{0}^d, \frac{\kappa }{\mathbf{r}} = \mathbf{w} \text { and }\mathbf{b} = (\frac{\mathbf{b}^{'}}{\mathbf{r}} - \mathbf{1}^d) \text { hold, then it goes}\\&\therefore \boxed {\mathbf{y} = \mathbf{w}\circ (\mathbf{t} - \mathbf{h})+\mathbf{b}}.\\ \end{aligned}$$

## Appendix C: Main component of RosE$_{2l}$

This derivation mainly involves certain elementary transformations based on the additive compositionality assumption. In this derivation, $\mathbf{w}_1$ and $-\mathbf{w}_2$ are the weight vectors of entity pair and relation embeddings that can rotate and scale them to fill the gap between expected relation embedding $\mathbf {r'}$ and real relation embedding $\mathbf{r}$. The quantities $\mathbf{b}$, $\mathbf{b}_1$, and $\mathbf{b}_2$ are *d*-dimensional bias vectors. $\mathbf{x}{1}$ is the difference between tail embedding $\mathbf{t}$ and head embedding $\mathbf{h}$ while vector $\mathbf{x}_2$ is equal to relation embedding $\mathbf{r}$; thus they are also *d*-dimensional vectors.

$$\begin{aligned}&\text {According to the additive compositionality principle, it goes}\\&\because \mathbf{h} + \mathbf{r} \approx \mathbf{t}, \\&\therefore \mathbf{t} - \mathbf{h} \approx \mathbf{r}.\\&\text {Weights }\mathbf{w}_{1} \text { and } -\mathbf{w}_{2} \text { are used for rotating and scaling, }\\&\therefore \mathbf{w}_{1}\circ (\mathbf{h} - \mathbf{t}) = -\mathbf{w}_{2}\circ \mathbf{r}. \\&\text {Further, it can be transformed equivalently,}\\&\therefore \mathbf{0}^d = \mathbf{w}_{1}\circ (\mathbf{h} - \mathbf{t})+\mathbf{w}_{2}\circ \mathbf{r}.\\&\text {Let vector }\mathbf{y}\text { denote the sum between }\mathbf{w}_{1}\circ (\mathbf{h} - \mathbf{t})\text { and }\mathbf{w}_{2}\circ \mathbf{r},\\&\therefore \mathbf{y} = \mathbf{w}_{1}\circ (\mathbf{h} - \mathbf{t})+\mathbf{w}_{2}\circ \mathbf{r}.\\&\text {If the conditions } \mathbf{y} = \mathbf{y}_{1} + \mathbf{y}_{2} \text { and } \mathbf{0}^d = \mathbf{b}_{1} + \mathbf{b}_{2}\text { hold, then}\\&\because \mathbf{y} = \mathbf{w}_{1}\circ (\mathbf{t} - \mathbf{h}) + \mathbf{w}_{2}\circ \mathbf{r},\\&\therefore \mathbf{y}_{1} + \mathbf{y}_{2} = \mathbf{w}_{1}\circ (\mathbf{t} - \mathbf{h}) + \mathbf{b}_{1} + \mathbf{w}_{2}\circ \mathbf{r} + \mathbf{b}_{2}.\\&\text {As a result, it can be decomposed as two linear functions,}\\&\therefore \mathbf{y}_{1} = \mathbf{w}_{1}\circ (\mathbf{t} - \mathbf{h}) + \mathbf{b}_{1}\text { and }\mathbf{y}_{2} = \mathbf{w}_{2}\circ \mathbf{r} + \mathbf{b}_{2}.\\&\text {If } (\mathbf{t} - \mathbf{h}) = \mathbf{x}_{1} \text { and }\mathbf{r}=\mathbf{x}_{2}\text { hold, then}\\&\therefore \boxed {\mathbf{y}_{1} = \mathbf{w}_{1}\circ \mathbf{x}_{1} + \mathbf{b}_{1}}\text { and }\boxed {\mathbf{y}_{2} = \mathbf{w}_{2}\circ \mathbf{x}_{2} + \mathbf{b}_{2}}\\ \end{aligned}$$

Notably, although the transformation follows certain conditions such as $\mathbf{y} = \mathbf{y}_{1} + \mathbf{y}_{2}$ and $\mathbf{0}^d = \mathbf{b}_1 + \mathbf{b}_2$, the intermediate variables $\mathbf{y}_1$, $\mathbf{y}_2$, $\mathbf{b}_1$ and $\mathbf{b}_2$ all disappear after some steps.
